# In Vitro Permeability Study of Homotaurine Using a High-Performance Liquid Chromatography with Fluorescence Detection Pre-Column Derivatization Method

**DOI:** 10.3390/molecules28207086

**Published:** 2023-10-14

**Authors:** Marianna Ntorkou, Eleni Tsanaktsidou, Konstantina Chachlioutaki, Dimitrios G. Fatouros, Catherine K. Markopoulou

**Affiliations:** 1 Laboratory of Pharmaceutical Analysis, Department of Pharmacy, Aristotle University of Thessaloniki, 54124 Thessaloniki, Greece; marianna.ntorkou98@gmail.com (M.N.); etsanaktsi@pharm.auth.gr (E.T.); 2 Laboratory of Pharmaceutical Technology, Department of Pharmacy, Aristotle University of Thessaloniki, 54124 Thessaloniki, Greece; kchachlioutaki@hotmail.com (K.C.); dfatouro@pharm.auth.gr (D.G.F.)

**Keywords:** homotaurine, derivatization, HPLC–FLD, CNS permeability study

## Abstract

Homotaurine (HOM) is considered a promising drug for the treatment of Alzheimer’s and other neurodegenerative diseases. In the present work, a new high-performance liquid chromatography with fluorescence detection (HPLC–FLD) (λex. = 340 nm and λem. = 455 nm) method was developed and validated for the study of substance permeability in the central nervous system (CNS). Analysis was performed on a RP-C18 column with a binary gradient elution system consisting of methanol–potassium phosphate buffer solution (pH = 7.0, 0.02 M) as mobile phase. Samples of homotaurine and histidine (internal standard) were initially derivatized with ortho-phthalaldehyde (OPA) (0.01 M), N-acetylcysteine (0.01 M) and borate buffer (pH = 10.5; 0.05 M). To ensure the stability and efficiency of the reaction, the presence of different nucleophilic reagents, namely (a) 2-mercaptoethanol (2-ME), (b) N-acetylcysteine (NAC), (c) tiopronin (Thiola), (d) 3-mercaptopropionic acid (3-MPA) and (e) captopril, was investigated. The method was validated (R^2^ = 0.9999, intra-day repeatability %RSD < 3.22%, inter-day precision %RSD = 1.83%, limits of detection 5.75 ng/mL and limits of quantification 17.43 ng/mL, recovery of five different concentrations 99.75–101.58%) and successfully applied to investigate the in vitro permeability of homotaurine using Franz diffusion cells. The apparent permeability (P_app_) of HOM was compared with that of memantine, which is considered a potential therapeutic drug for various CNSs. Our study demonstrates that homotaurine exhibits superior permeability through the simulated blood–brain barrier compared to memantine, offering promising insights for enhanced drug delivery strategies targeting neurological conditions.

## 1. Introduction

Alzheimer’s and Parkinson’s diseases are the two most prevalent neurodegenerative disorders. The etiology of these conditions is multifactorial, involving complex biological pathways. The two most common mechanisms for the development of Alzheimer’s disease are the deposition of amyloid beta-protein plaques in the brain and mutations in the tau-protein gene [1]. Both mechanisms lead to the formation of neurofibrillary tangles, causing dysfunction in the transmission of nerve signals in the brain. Current therapeutic strategies for Alzheimer’s disease include the use of memantine, a N-methyl-D-aspartic acid receptor antagonist and specific cholinesterase inhibitors (rivastigmine, galantamine and donepezil) [1]. However, recent clinical research has shifted its focus to homotaurine as a potential candidate for future therapy [2,3].

Homotaurine, initially extracted from various algae species [4], has been chemically synthesized and utilized in clinical practice. It exhibits specific anti-amnestic activity [5] and high affinity for the gamma-aminobutyric acid receptor in the brain [6]. Homotaurine is commercially available in dietary supplements and over-the-counter medications. Moreover, it has been proposed for the treatment of Parkinson’s disease to prevent cognitive decline [7]. Due to its neuroprotective properties, homotaurine has been studied as a memory enhancer in preclinical studies and Phase II and III trials [3]. However, homotaurine has not yet received FDA approval as a drug.

**Scheme 1:**
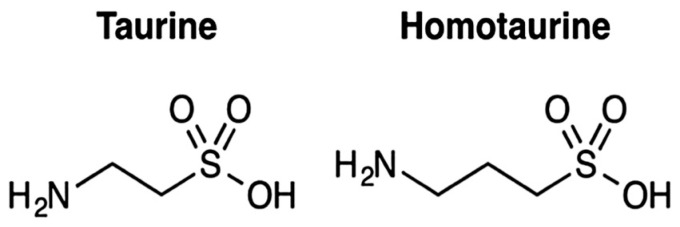
Chemical structures of taurine and homotaurine.

Structurally (Scheme 1), homotaurine is a naturally occurring small amino-sulfonate compound that shares structural similarities with the amino acid taurine. The quantification and chromatographic separation of taurine have been extensively studied using gas chromatography [8,9], ion chromatography [10], capillary electrophoresis [11] and HPLC [12]. Similarly, homotaurine has been identified as an impurity of the calcium acamprosate via electrophoresis [13], and its concentration in blood has been determined using HPLC–ELSD (Evaporative Light Scattering Detection) [14]. It has also been identified in rat plasma through pre-column derivatization with OPA using HPLC and fluorescence detection [15]. However, these techniques have limitations in sensitivity, making them unsuitable for further clinical studies (Phase I or II). Mass spectrometry, although expensive, is recommended as the method of choice in such cases [16]. Fluorescence is also considered a reliable, economical and highly sensitive technique and can be used provided that the substance under examination fluoresces. Alternatively, the analyte is reacted with a fluorescent reagent, such as 2,4-dinitrofluorobenzene [17], Dansyl-Cl (for primary and secondary amino acids) [18] and fluorescein [19] by the derivatization process. One potential drawback is the relatively long reaction time required, which usually takes place under special conditions (e.g., high temperature, stirring), and the instability of the resulting derivatives. Ortho-phthalaldehyde (OPA) is a widely used reagent for the derivatization of substances containing primary amino groups in their molecule, as it reacts rapidly and selectively (under alkaline conditions) to form strongly fluorescent derivatives. The reaction is conducted in the presence of a thiol-reducing reagent, such as 2-mercaptoethanol, urea and sodium sulfite [20], N-acetyl-L-cysteine [21] and 3-mercaptopropanoic acid [16]. In such cases, preliminary research is deemed necessary to select a stable and effective OPA derivative.

Franz cells have primarily been used for the study of drugs intended for transdermal or topical administration, providing reliable and repeatable results. These vertical static glass diffusion cells are commonly employed to assess the in vitro permeability of substances. It is considered an economical and “green” technique, as it requires small drug volume and a minimal number of membranes. The in vitro permeability of molecules across the blood–brain barrier has mainly been studied using the PAMPA test (Parallel Artificial Membrane Permeation Assay), through an artificial biomimetic membrane impregnated with a phospholipid solution [22,23]. In order to simplify the biomimetic setup, researchers have previously adopted the Franz diffusion cells by utilizing membranes derived from the PAMPA technique in a study of intestinal epithelial membranes. This modified method is referred to as Franz-PAMPA [23].

The aim of the present experimental effort was to develop a sensitive, flexible and reliable HPLC–FLD method appropriate for the determination of homotaurine in permeability studies in Franz cells.

To fulfill the purpose, the performance and stability of homotaurine derivatives with (a) 2-mercaptoethanol (2-ME), (b) N-acetylcysteine (NAC), (c) tiopronin (Thiola), (d) 3-mercaptopropionic acid (3-MPA) and (e) captopril were initially examined. Subsequently, an appropriate HPLC–FLD analytical method for quantifying homotaurine was developed and validated. Finally, the method was employed for the in vitro permeability study of the substance across the blood–brain barrier using a Franz cell arrangement. The results showed high precision and accuracy and were correlated with those obtained from memantine, which is considered as the drug of choice for CNS diseases.

## 2. Results and Discussion

### 2.1. Optimization of the Derivatization Reaction

**Figure 1:**
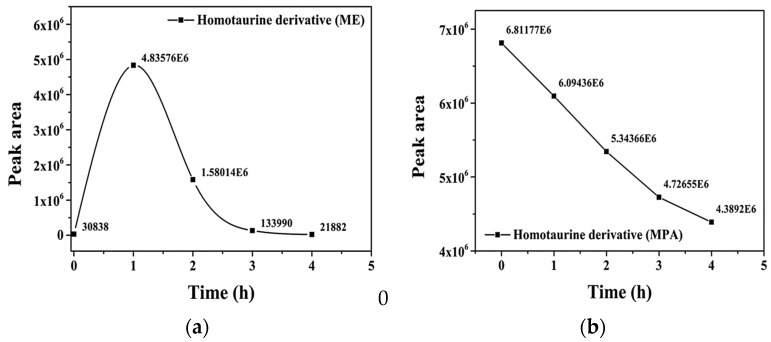

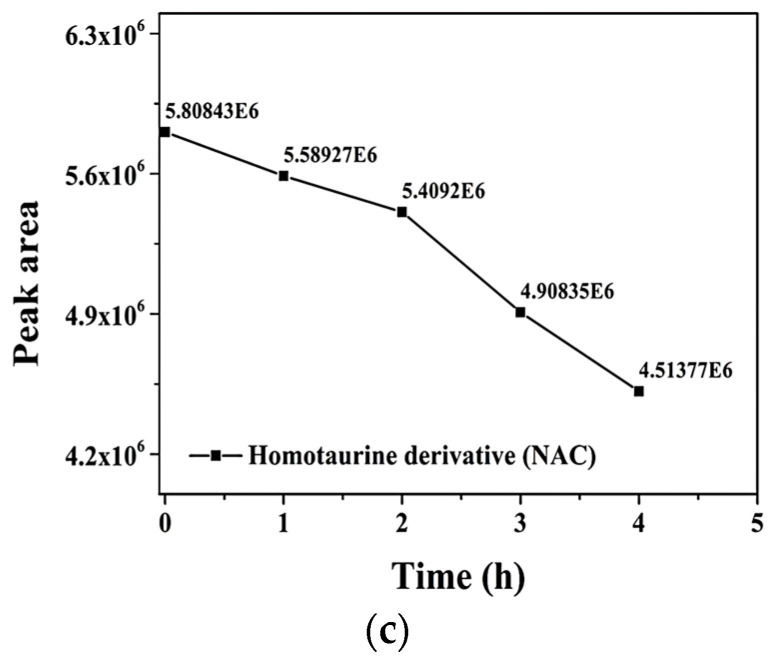
Stability study of homotaurine derivative in the presence of: (**a**) 2-ME; (**b**) 3-MPA; (**c**) NAC.

The stability of the derivatization product provided by the five nucleophiles (0.5 μg/mL) was first studied over a period of 0–4 h (Figure 1a–c). According to the results, the 2-ME derivative shows limited stability. In particular, an increase in reaction kinetics was observed in the first hour, suggesting that the reagent needs some time to react. However, within 4 h, the 2-ME derivative shows complete decomposition (99.55%). Next, the stability of the 3-MPA complex was also examined. Although the 3-MPA derivative shows a high signal intensity, after 4 h, its signal decreased by 35.56%. In the case of tiopronin, despite the investigation of various parameters that could influence the reaction, no derivative formation was observed. Therefore, the reagent was discarded. For captopril, two adjacent peaks were identified in the chromatogram. The area of the first peak decreased over time, while the area of the second increased. Due to the proven instability of the complex, further investigation was deemed inappropriate. Finally, when examining NAC, it was found that although it presents a slightly lower peak area, compared to the 3-MPA derivative, it is more stable (22.28% degradation). Therefore, due to its therapeutic and non-toxic properties, as well as environmental friendliness, NAC was selected as the most suitable reducing agent.

**Figure 2:**
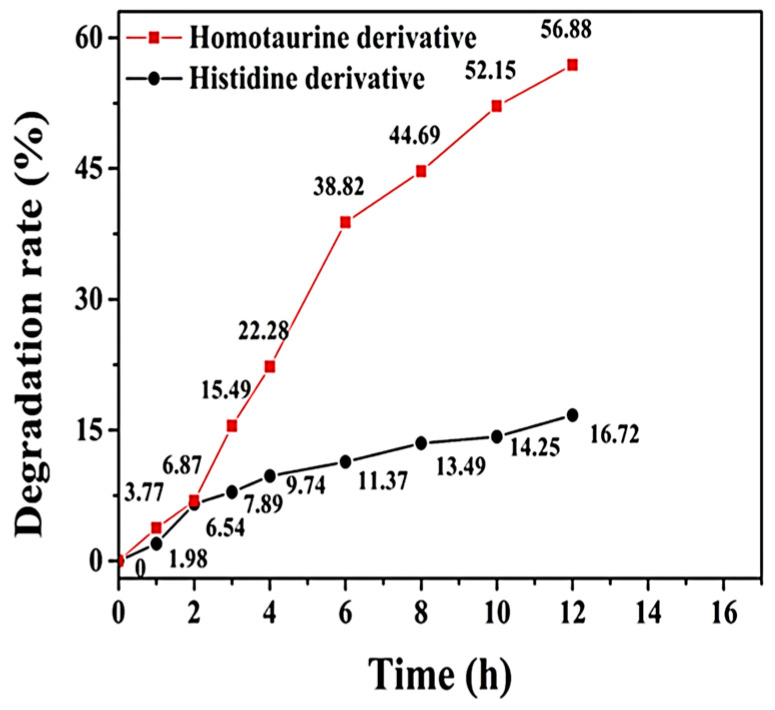
Stability study of homotaurine and histidine derivatives in the presence of NAC.

Moreover, an extensive stability study of homotaurine and histidine (internal standard) derivatives with NAC and OPA was caried out over 12 h. As shown in Figure 2, both substances exhibit similar behavior in the first two hours, but their behavior rates diverge afterward. Therefore, for results to be comparable, it is important to prepare fresh samples and analyze them immediately (within 2 h).

**Figure 3:**
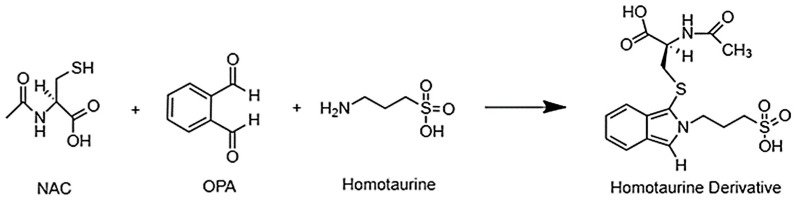
Derivatization reaction of homotaurine in presence of NAC.

The method of the derivatization of amino acids with OPA and thiols was extensively studied by Ankhbayar and Hwan-Ching [24], who report in their work the complete organic reaction mechanism. In our case, to further optimize the method (Figure 3), additional parameters (OPA concentration, time, pH) that could affect the reaction were investigated. During the experiments, all parameters were kept constant and only the one studied was varied. The responses to evaluate the results were mainly the HOM peak area and the good appearance of the chromatograms. First, as the addition of different volumes (50, 100 and 200 μL) of OPA (0.01 M) does not significantly improve the results (Table 1), the volume of 100 μL OPA was considered optimal in order to avoid by-products and prevent column overload.

Regarding the selection of the appropriate buffer, the use of a borate solution is recommended since, due to its high buffering capacity (pH 8.0–10.0), it prevents the ionization of secondary amino groups [21]. Under these conditions, one of the most important factor to be studied was pH (Table 2) in the range of 8.5–11.0. A value of 9 was chosen as optimal since derivatization was assured and the chromatogram was improved (lower tailing factor). Then, the time required for the reaction was also critical [21] and was investigated between 0 and 30 min (Table 3). Based on the results, it was observed that the derivatization is completed immediately, and up to about 30 min, the HOM derivative is relatively stable. Furthermore, for practical and reproducibility reasons, it was decided to analyze the samples exactly 10 min after their preparation.

Summarizing the procedure, the following steps are included: 100 μL homotaurine working standard solution was mixed with 100 μL OPA (0.01 M), 100 μL NAC (0.01 M), 700 μL borate buffer (pH 10.5; 0.05 Μ) and 100 μL of histidine (internal standard) in a clean glass HPLC vial. The ingredients were vortexed for 10 s and the derivatization mixture was allowed to react for 10 min at room temperature before injected for analysis in HPLC. At the same time, blanks were prepared by substituting homotaurine and histidine with deionized water.

### 2.2. Optimization of Chromatographic Method

Although taurine could be considered as a suitable internal standard, it was rejected because the substance co-eluted with homotaurine or other impurities. Therefore, since homotaurine belongs to amino acids, alanine and histidine (with carboxyl groups instead of sulfonate) were also examined. Alanine presents similar chromatographic behavior to taurine and was discarded. Thus, histidine was the most appropriate internal standard as it reacts quantitatively with OPA and NAC and presents good chromatographic separations.

Furthermore, several critical parameters that could affect the quality of the chromatogram were investigated and optimized (the stationary phase, the elution strength of the mobile phase, buffer pH and concentration, column flow rate and temperature, injection volume, the elution program and the detection wavelengths).

In order to choose the appropriate stationary phase with the best performance, the cyan, amino and C_18_ columns were tested under the same chromatographic conditions (mobile phase: phosphate buffer 0.02 M-methanol, pH 7.0). Using both cyan and amino columns, in most cases, HOM eluted with the solvent front or did not provide separation. Thus, C_18_ was selected for further experiments as it was the only column that met the analytical requirements.

Acetonitrile and methanol were tested as organic solvents for the mobile phase, either in binary or ternary mixtures with water. Since their effect was similar, methanol was chosen as the most suitable, and the optimum methanol/water ratio (22:78, *v*/*v*) was determined. Further improvements in the mobile phase were made by replacing the water with an aqueous buffer.

Typically, for amino acid or homotaurine analysis, phosphate [16], ammonium acetate [16] and sodium acetate [25] buffers are recommended. In our case, potassium phosphate was used as it exhibits good buffer capacity and low background signal. Three different pH values (equal to 3.0, 5.0 and 7.0) of the buffer solution were studied (Table 4). In all cases, differences in peak width and resolution were observed. The decrease in the elution time of the HOM derivative, at lower pH values, is due to the ionization of its amino group since both the carboxylic and sulfonic moieties are permanently ionized. Among the pH values studied, 7.0 was chosen as optimal as it improves both the tailing factor and the resolution, as well as ensuring the stability of HOM. 

Additionally, different values of 0.02, 0.04 and 0.06 M potassium phosphate solution (at pH = 7.0) were used to study the effect of buffer concentration. According to the results, no significant differences were observed regarding peak areas and retention times (Table 5). Thus, the lowest concentration of 0.02 M was chosen to avoid column overload and backpressure problems. It is generally known that increasing the column temperature results in a decrease in the elution time of analytes. However, this may lead to incomplete separation or a negative effect on the fluorescence signal [25]. Considering all the data, temperatures ranging from 25 to 35 °C were tested (Table 6) and 30 °C was selected as ideal since it provides a higher peak area for HOM and improves the tailing factor and the selectivity.

Finally, a gradient elution program was considered necessary to achieve short total run time, adequate separations as well as impurities disposal. Therefore, potassium phosphate buffer solution (pH = 7.0; 0.02 Μ) was chosen as mobile phase A, while methanol as B. Initially, for up to 8 min, the ratio of B was 22% *v/v,* and in the next 2 min, was linearly increased to 40%. Then, within 10 min, the system was reverted to its initial conditions (22% B) and kept constant for up to 5 min to obtain reproducible separations.

According to similar studies, amino acid derivatives have been mainly monitored at excitation wavelengths of 330 or 340 nm and at emissions of 450 or 455 nm [16,25,26]. Aiming to achieve maximum signal, the detector was set at 340 nm (λ_ext_) and 455 nm (λ_em_), respectively. 

Finally, the sample injection volume was studied in the range of 10 to 50 µL. The procedure was carried out at two concentration levels of homotaurine (0.05 and 5 μg/mL). Low concentrations were tested to ensure the adequacy of analytical signal, under extreme conditions, while the higher concentrations were tested to ensure the separation of homotaurine (tr = 8.5 min) from a neighboring interference peak (tr = 6.8 min). Therefore, 40 µL was chosen as the optimal injection volume.

### 2.3. In Vitro Permeability Study

Our findings indicate that homotaurine exhibits a higher permeation rate through the simulated BBB model when compared to memantine. This phenomenon is consistent with the established characteristics of these compounds. Homotaurine, with its lower molecular weight [27] and lower lipophilicity [28], offers favorable attributes for permeation across biological barriers. In contrast, memantine, classified as a BCS class III compound with high solubility but low permeability, exhibits a relatively lower permeation rate. Such differences in the physicochemical properties of the two compounds justify the observed variation in their penetration. Corresponding observations and results were reported in a study by Takagi et al. [29], where they highlight the different penetration profiles of memantine and compounds with similar characteristics. The consistency between our results and those reported in the literature strengthens our confidence in the methodology used. Importantly, the increased penetration profile of homotaurine, compared to memantine, emphasizes the potential advantages of HOM in drug delivery applications targeting the central nervous system.

### 2.4. Method Validation

The selectivity, linearity, limits of detection (LOD) and quantification (LOQ), the precision and accuracy of the analytical method were studied to conduct the validation guideline according to ICH Q2(R2) [30]. 

#### 2.4.1. Selectivity

**Figure 4:**
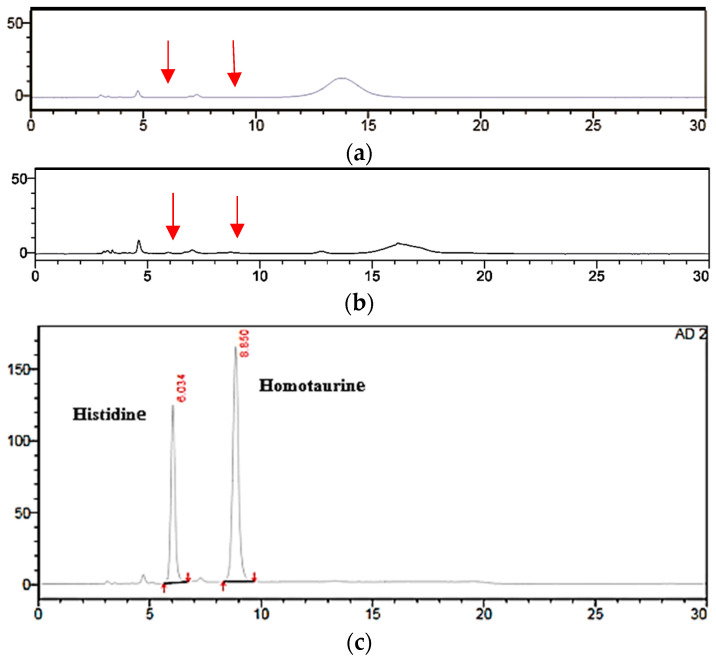
Representative chromatogram of: (**a**) blank of the reaction; (**b**) blank of PBS matrix and (**c**) spiked sample with homotaurine (45.5 ng/mL, t_R_ = 6.034 s) and histidine (45.5 ng/mL, t_R_ = 8.850 s). Red arrows indicate the time points at which the histidine and homotaurine derivatives are eluted, at 6.034 and 8.850 s, respectively. At these time points, there are no interfering peaks at the two blank chromatograms.

For selectivity, both blank (of the reaction and of PBS matrix) and sample (spiked with a given concentration of the substance and internal standard) chromatograms were compared. According to Figure 4a–c, the peaks of the blank do not interfere with those of the standard, which proves the selectivity of the method. 

#### 2.4.2. Linearity

Homotaurine standard solutions were prepared at seven concentration levels (Table 7). A total volume of 100 μL of each level were spiked with 100 μL of 0.5 μg/mL histidine solution, followed by their derivatization reaction to a volume of 1.1 mL. The final concentrations of the standard solutions along with the statistical results of the calibration curve are presented in Table 1.

#### 2.4.3. Precision

The repeatability of the method within the same day (intra-day repeatability) and the intermediate precision (inter-day precision, for three consecutive days) were examined. To ensure repeatability over the entire concentration range of the calibration curve, three concentration levels of homotaurine standard solutions were tested: the lowest (4.55 ng/mL), the intermediate (45.45 ng/mL) and the highest (454.55 ng/mL). For each concentration, three replicates were performed, and the average responses, standard deviation (SD) and relative standard deviation (%RSD) were calculated. Additionally, for the intermediate precision, the mean of the relative standard deviations of the three concentration levels for each day was calculated, as well as the mean of the relative standard deviations of the three days (Table 8).

#### 2.4.4. Accuracy

The accuracy of the proposed method was investigated in two ways: (a) by analyzing five samples of (blank-matrix) spiked with 4.55 ng/mL HOM and (b) by analyzing (after derivatization) five samples of the substance at five different concentration levels. Three individual replicates were performed for each sample. The evaluation of the proposed method is based on %Recovery and %RSD results (Table 8). Confidence limits were set using *t*-test statistics (*p* = 0.05 and 4 degrees of freedom).

#### 2.4.5. Limit of Detection (LOD) and Limit of Quantification (LOQ)

The detection and quantification limits of the method were calculated according to the signal-to-noise ratio approach proposed by ICH Q2(R2) Guideline [30]. The limits of detection and quantitation were found as 0.455 and 1.365 ng/mL, respectively. 

### 2.5. In Vitro Permeability Study

According to recent experimental research, diffusion through Franz cells and PAMPA (Parallel Artificial Membrane Permeability Assay) membranes may provide an alternative technique for studying other biological barriers, such as the blood–brain barrier, skin and mucosal tissues (e.g., buccal or nasal) [23,31]. Therefore, in the present study, the Franz cell apparatus and a specially modified lipophilic membrane were used to simulate blood–brain barrier permeability.

After the experiment was completed, the amount (μg) of homotaurine and memantine that passed through the membrane into the receptor compartment (20 mL), at different time points, was calculated for each cell and expressed as μg/cm^2^. The steady-state flux parameter (J_ss_) was also calculated based on the slope of the line plot of drug permeation (μg/cm^2^) versus time (h) (Table 9). Finally, the P_app_ permeability coefficient of the substances was calculated (Table 9) using the following Equation (1):

P_app_ = J_ss_/C_o_, (1)

where J_ss_ is the steady-state flux (μg/cm^2^/h) andC_o_ (μg/mL) is HOM concentration in the donor compartment.

**Figure 5:**
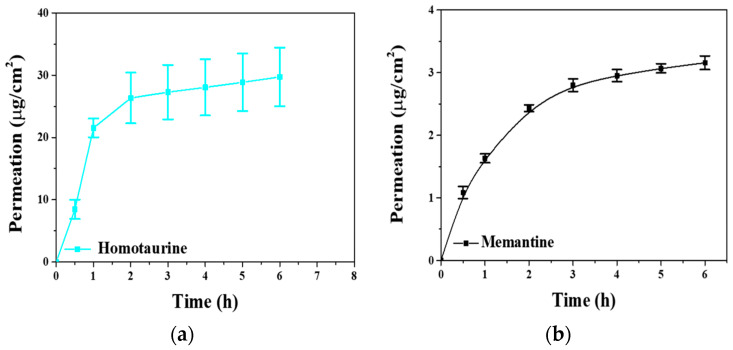
(**a**) Permeability study of homotaurine; (**b**) Permeability study of memantine.

Figure 5 illustrates the cumulative penetration of substances into the receptor compartment at different sampling intervals. According to the results (Figure 5a,b), after 3 h, almost constant amounts of homotaurine and memantine diffuse through the membrane. When comparing the two substances, it is evident that homotaurine diffuses to a greater extent than memantine at the same time points. The primary reason for this behavior can be attributed to the disparate molecular weights of the two substances (homotaurine weighing 139.17 g/mol and memantine weighing 179.3 g/mol). Additionally, there are differences in molecular volume, expressed as refractivity (54.49 m^3^·mol^−1^ for memantine, 29.47 m^3^·mol^−1^ for homotaurine) and lipophilicity (memantine logP = 3.31, homotaurine logP = −2), which could also contribute to this discrepancy. Therefore, homotaurine permeates more easily through the membrane, as shown by the J_ss_ and P_app_ values, respectively (Table 9).

## 3. Materials and Methods

### 3.1. Chemicals, Materials and Reagents

For HPLC analysis, acetonitrile (ACN) and methanol (MeOH) were of analytical grade and were purchased from VWR Chemicals (Radnor, PA, USA), while the water was of high purity (18.2 resistivity MΩ cm), produced by a B30 purification system (Adrona SIA, Riga, Latvia). A potassium phosphate buffer (pH 7.0; 0.02 M) was prepared for the mobile phase by dissolving 2.72 g of KH_2_PO_4_ (Sigma-Aldrich, Steinheim, Germany) in 1L distilled water. The pH was adjusted by adding a NaOH solution 0.1 M. 

For the derivatization reaction, 0.01 M stock solutions of o-phthalaldehyde (OPA) (Sigma-Aldrich, Steinheim, Germany), N-acetyl-L-cysteine (NAC) (Sigma-Aldrich, St. Louis, MO, USA), 2-mercaptoethanol (2-ME) (Ega-Chemie, Steinheim, Germany), 3-mercaptopropionic acid (3-MPA) (Sigma Aldrich, St. Louis, MO, USA), tiopronin (Thiola) (Sigma-Aldrich, Steinheim, Germany) and captopril (Sigma-Aldrich, Steinheim, Germany) were prepared. The OPA reaction occurs under alkaline conditions to prevent ionization of secondary amino groups, so a borate buffer (pH 10.5; 0.05 Μ) (Merck, Darmstadt, Germany) was used as incubation medium.

For the calibration curve, standard stock solutions of histidine 0.5 μg/mL (Sigma-Aldrich, Steinheim, Germany) and homotaurine 100 μg/mL (Sigma-Aldrich, Steinheim, Germany) were prepared. Appropriate dilutions were made to obtain 7 levels of homotaurine concentration, ranging from 0.05 to 5 µg/mL. The samples underwent the derivatization process and analyzed via HPLC. 

For the in vitro permeability study of homotaurine using Franz diffusion cells, a phosphate-buffered saline solution (PBS) (pH 7.4) was prepared by dissolving 8.0 g NaCl, 1.44 g Na_2_HPO_4_, (Merck, Darmstadt, Germany), 0.24 g KH_2_PO_4_ (Sigma-Aldrich, Steinheim, Germany) and 0.20 g KCl (Chem-Lab NV, Zedelgem, Belgium) in 1 L of distilled water.

### 3.2. Derivatization Procedure

The signal intensity and the stability of HOM derivatives with different nucleophiles’ reagents were tested over a period of 0 to 4 h. Therefore, N-acetyl-L-cysteine (NAC), 2-mercaptoethanol (2-ME), 3-mercaptopropionic acid (3-MPA), tiopronin (Thiola) and captopril were studied. Among them, the stability of the most suitable reagent (reaction with NAC) was further studied over a period of 24 h. 

After investigating the reaction conditions (pH, time, OPA concentration), the final derivatization process (Figure 2) was as follows: 100 μL homotaurine solution was mixed with 100 μL OPA (0.01 M), 100 μL NAC (0.01 M), 700 μL borate buffer (pH 10.5; 0.05 Μ) and 100 μL of histidine (internal standard) in glass HPLC vial. After vortexed for 10 s, the derivatization mixture was left to react for 10 min at room temperature and injected as it is in HPLC–FLD.

### 3.3. Permeability Process in the Franz Diffusion Cells

A 20 mL Franz cell assembly (2.5 cm diameter) consisting of two chambers (donor and acceptor) was used for CNS permeability experiments. A cellulose membrane modified with a lipid solution of isopropyl myristate [32] was placed between the two chambers to simulate the lipid composition of the blood–brain barrier. Phosphate-buffered saline (PBS) adjusted to pH 7.4 was used as the solution in the donor and receptor chambers. The temperature was maintained at 37 °C, by circulating hot water in the double-walled outer enclosure of the chamber, and gentle agitation at 70 rpm was applied.

The donor was initially injected (t = 0) with 2 mL of homotaurine solution (100 μg/mL). Samples of 0.5 mL were collected from the receptor compartment at the 0.5, 1, 2, 3, 4, 5 and 6 h time points, and immediately replaced with an equal volume of freshly prepared and pre-warmed PBS solution at 37 °C. The process was repeated three times, while a fourth cell was used as a “blank”. Samples were derivatized and analyzed using HPLC. The same process was eventually repeated for memantine. Memantine samples were analyzed using a validated method proposed by Zarghi and co-workers [33].

### 3.4. Instrumentation and Chromatographic Conditions

Chromatographic separation of homotaurine and histidine was performed using a Shimadzu HPLC system consisting of two LC-20AD isocratic pumps, a DGU-14A degasser, an SIL-10AD autosampler and a fluorescence detector (RF-535, Shimadzu, Tokyo, Japan). The stationary phase was a reversed-phase LC-C_18_ DB column (250 × 4.6 mm, 5.0 μm), Supelco (Bellefonte, PA, USA), thermostated at 30 °C. LC-solution^®^ software version 1.25 SP4 was used for hardware control and data manipulation. All separations were performed using a binary gradient elution program. Mobile phases A and B consisted of potassium phosphate buffer (pH = 7; 0.02 M) and methanol, respectively. The injection volume was set at 40 µL and the flow rate was set at 1 mL·min^−1^. Homotaurine and histidine OPA derivatives were monitored at λ_ext_ = 340 and λ_em_ = 455 nm, respectively.

## 4. Conclusions

An analytical method was developed for the accurate quantification of homotaurine via HPLC and a fluorescence detector. The analysis of both homotaurine and histidine (internal standard) was performed after derivatization with OPA, ACA and borate buffer. The procedure was validated (based on ICH guidelines) ensuring linearity, precision and accuracy. Finally, the proposed method was successfully used to investigate the in vitro permeability of homotaurine through the blood–brain barrier using Franz cells.

## Figures and Tables

**Table 1:** Effect of OPA concentration on reaction efficiency.

	50 μL		100 μL		200 μL	
	Homotaurine	Histidine	Homotaurine	Histidine	Homotaurine	Histidine
Retention time	6.778	5.159	6.658	5.041	6.772	5.149
Peak area	9,291,676	3,302,120	9,170,223	3,497,763	8,922,560	3,237,679
Tailing factor	2.795	1.990	2.395	1.976	2.325	1.937

**Table 2:** Influence of the pH.

	pH = 8.5		pH = 9	
	Homotaurine	Histidine	Homotaurine	Histidine
Retention time	7.144	5.208	7.766	5.534
Peak area	9,745,111	3,490,905	8,962,345	3,216,106
Tailing factor	2.048	2.100	1.852	1.724
	pH = 10.5		pH = 11	
	Homotaurine	Histidine	Homotaurine	Histidine
Retention time	6.658	5.041	6.721	5.111
Peak area	9,170,223	3,497,763	9,071,322	2,224,509
Tailing factor	2.415	1.976	2.721	1.931

**Table 3:** Effect of time on the kinetics of the derivatization reaction.

	t = 0 min		t = 5 min	
	Homotaurine	Histidine	Homotaurine	Histidine
Retention time	6.635	5.045	6.539	5.006
Peak area	9,329,892	2,760,075	8,994,530	3,337,056
Tailing factor	2.660	2.034	2.599	1.985
	t = 10 min		t = 30 min	
	Homotaurine	Histidine	Homotaurine	Histidine
Retention time	6.567	4.999	6.526	5.012
Peak area	9,069,873	3,189,284	8,851,468	3,268,445
Tailing factor	2.689	1.952	2.863	1.983

**Table 4:** Effect of mobile-phase pH.

	pH = 3		pH = 5		pH = 7	
	Homotaurine	Histidine	Homotaurine	Histidine	Homotaurine	Histidine
Retention time	6.506	4.888	8.166	5.497	8.552	5.911
Peak area	804,412	210,781	9,368,871	2,069,597	9,442,442	3,918,684
Tailing factor	1.477	1.158	2.029	2.491	1.920	2.264
Resolution	1.795		5.203		4.365	

**Table 5:** Influence of the phosphate buffer concentration.

	C = 20 mM		C = 40 mM		C = 60 mM	
	Homotaurine	Histidine	Homotaurine	Histidine	Homotaurine	Histidine
Retention time	7.766	5.534	8.079	5.745	8.552	5.911
Peak area	8,962,345	3,216,106	8,952,314	3,096,572	9,463,550	3,124,075
Tailing factor	1.852	1.724	1.887	1.873	1.845	1.753

**Table 6:** Effect of the column temperature.

	T = 25 °C		T = 30 °C		T = 35 °C	
	Homotaurine	Histidine	Homotaurine	Histidine	Homotaurine	Histidine
Retention time	7.270	5.200	6.757	4.962	6.068	4.683
Peak area	9,442,442	3,918,684	9,653,686	3,616,461	9,176,446	2,671,120
Tailing factor	1.920	2.491	1.722	2.161	2.438	1.965
Resolution	4.365		3.824		2.914	

**Table 7:** Results of linearity, regression analysis, LOD and LOQ.

Linearity							
C_initial_ (μg/mL)	0.05	0.075	0.1	0.5	1	2	5
C_final_ (ng/mL)	4.55	6.82	9.09	45.45	90.91	181.82	454.55
Regression Equation				Y = 0.033x + 0.1108			
R^2^				0.9999			
Slope				0.033			
Standard Error				0.05750			
LOD (ng/mL)				0.455			
LOQ (ng/mL)				1.365			

**Table 8:** Results of accuracy and precision.

C_theoritical_ (ng/mL)	C_experimental_ (ng/mL)	% Recovery	Mean % Recovery	Standard Deviation	Limits of Confidence
4.55	4.61	101.58	101.33	1.03	101.33 ± 1.28
45.45	46.57	102.47			
90.91	92.59	101.85			
181.82	183.60	100.98			
454.55	453.44	99.75			
C_theoritical_ (ng/mL)	C_experimental_ (ng/mL)	% Recovery	Mean % Recovery	Standard Deviation	
4.55	4.76	104.8	98.7	6.5	
4.55	4.63	101.8			
4.55	5.10	101.5			
4.55	4.00	88.0			
4.55	4.43	97.4			
Intra-day and Inter-day precision					
Day 1					
Concentration level (ng/mL)		4.55	45.45	454.55	
% RSD		2.0%	1.1%	1.4%	
Mean % RSD		1.5%			
Day 2					
Concentration level (ng/mL)		4.55	45.45	454.55	
% RSD		3.5%	1.6%	2.9%	
Mean % RSD		2.7%			
Day 3					
Concentration level (ng/mL)		4.55	45.45	454.55	
% RSD		6.9%	1.7%	0.98%	
Mean % RSD		3.2%			
Overall of 3 days		2.5%			

**Table 9:** Steady-state flux J_ss_ and P_app_ coefficient calculation for homotaurine and memantine.

Cell	J (μg/cm^2^/h) Homotaurine	J (μg/cm^2^/h) Memantine	P_app_ (h/cm^2^) Homotaurine	P_app_ (h/cm^2^) × 10^−3^ Memantine
a	1.197	0.114	5.983	0.571
b	0.936	0.129	4.679	0.648
c	0.858	0.113	4.290	0.566
Mean	0.997	0.189	4.984	0.595
Standard Deviation	0.180	0.009	0.887	0.046

## Data Availability

Data is contained within the article.

## Sample Availability

Not available.
